# Bacterial serine protease HtrA as a promising new target for antimicrobial therapy?

**DOI:** 10.1186/s12964-017-0162-5

**Published:** 2017-01-10

**Authors:** Silja Wessler, Gisbert Schneider, Steffen Backert

**Affiliations:** 1 Department of Molecular Biology, Division of Microbiology, Paris-Lodron University of Salzburg, Billroth Str. 11, A-5020 Salzburg, Austria; 2 Department of Chemistry and Applied Biosciences, Swiss Federal Institute of Technology (ETH), Vladimir-Prelog-Weg 4, CH-8093 Zürich, Switzerland; 3 Division of Microbiology, University of Erlangen-Nuremberg, Staudtstr. 5, D-91058 Erlangen, Germany

## Abstract

Recent studies have demonstrated that the bacterial chaperone and serine protease high temperature requirement A (HtrA) is closely associated with the establishment and progression of several infectious diseases. HtrA activity enhances bacterial survival under stress conditions, but also has direct effects on functions of the cell adhesion protein E-cadherin and extracellular matrix proteins, including fibronectin and proteoglycans. Although HtrA cannot be considered as a pathogenic factor per se, it exhibits favorable characteristics making HtrA a potentially attractive drug target to combat various bacterial infections.

## Background

**Fig. 1:**
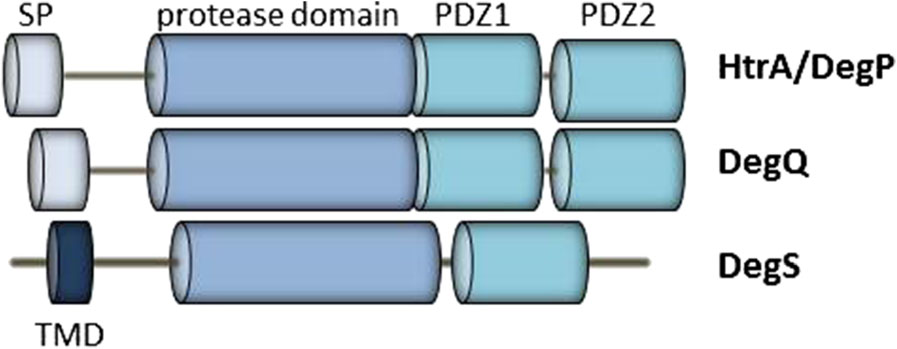
Domain structure of HtrA proteins in Gram-negative bacteria. Monomeric DegP and DegQ proteins harbor an N-terminal signal peptide (SP), an ATP-independent trypsin-like protease domain followed by two PDZ domains. Many DegS proteins are composed of a transmembrane domain (TMD), protease domain and one PDZ domain

HtrA proteins and their orthologues represent an important class of heat-shock-induced serine proteases and chaperones protecting protein structures. They are expressed in both prokaryotic and eukaryotic species, including plants and humans [1–3]. Whereas HtrA orthologues commonly display proteolytic activities against multiple target proteins, their structural architecture and physiological functions are rather miscellaneous and differ between species. In many bacteria, HtrA proteases are composed of an N-terminal signal peptide, followed by a trypsin-like serine protease domain and one or two C-terminal PDZ (postsynaptic density protein [PSD95], *Drosophila* disc large tumor suppressor [Dlg1], and zonula occludens-1 protein [ZO-1]) modules which permit intermolecular protein-protein interactions [4, 5] (Fig. 1). In Gram-negative bacteria, HtrA proteases are generally transported into the periplasm, where they form proteolytic active multimers with known functions in protein quality control. The best characterized HtrA proteins are the *Escherichia coli* DegP, DegQ, and DegS orthologues [6, 7]. All these different HtrAs display a high degree of sequence identity in their protease domain, but exhibit numerous specific features and activities [6]. DegP and DegQ harbor two PDZ domains, while DegS often contains a transmembrane domain and only one PDZ domain [1, 8] (Fig. 1). DegP is well characterized as a protease with ATP-independent chaperone functions. Its active oligomers assemble upon target binding and hydrolyze unfolded or misfolded proteins into small peptides [9, 10]. DegS represents a regulatory protease which cleaves the anti-sigma factor RseA, while the physiological functions of DegQ are not fully understood [11]. Inactivation of the *htr*A gene by mutation causes an increased sensitivity to stress, e.g., elevated temperature, of all bacteria investigated to date [12–18].

### (Patho)-physiological function of bacterial HtrA

Until recently, it has been commonly accepted that HtrA family members of bacteria are strictly acting inside the periplasm. However, we have recently unraveled a hitherto unknown function of HtrA during bacterial infection. *Campylobacter jejuni* and its close relative *Helicobacter pylori* actively secrete HtrA proteins in the extracellular environment, where they target host cell factors [19–21]. HtrA was also identified in outer membrane vesicles released by *C. jejuni*, *H. pylori*, *Vibrio cholera*, *Chlamydia muridarum* or *Borrelia burgdorferi* [22–26]. Infection experiments with polarized cell monolayers in vitro suggested that *H. pylori* and *C. jejuni* HtrA can disrupt the epithelial barrier by opening cell-to-cell junctions. This remarkable effect is achieved by cleaving-off the extracellular domain of the surface adhesion protein and tumor suppressor E-cadherin, and probably other junctional proteins by HtrA, followed by paracellular bacterial transmigration [20, 21]. The deletion of the *htrA* gene in *C. jejuni* led to a defect in E-cadherin shedding and causes impaired transmigration of the bacteria across monolayers of polarized epithelial cells in vitro [19, 21].

In particular, E-cadherin showed to be an important factor for establishing and maintaining epithelial integrity in the host. E-cadherin is a single transmembrane protein, which consists of an intracellular domain (IC), a transmembrane domain (TD), and five extracellular domains (EC) [27]. EC domains establish homophilic interactions in *cis* and *trans* that require calcium binding to the linker region between the EC domains. We have recently identified the cleavage sites of *H. pylori* HtrA in E-cadherin. Mass-spectrometry-based proteomics and Edman degradation revealed three signature motifs containing the [VITA]-[VITA]-x-x-D-[DN] sequence pattern as preferentially cleaved by HtrA [28]. The results of our studies also suggest that the presence of calcium ions blocks HtrA-mediated cleavage by interfering with the accessibility of calcium-binding regions between the individual EC domains harboring the HtrA cleavage sites [29]. Investigating *C. jejuni* Δ*htrA* deletion mutants in in vivo studies, it was demonstrated that HtrA plays a crucial role during infection by triggering host cell apoptosis and immunopathology in mice [30, 31]. Similarly, HtrA is critical for the virulence of many other pathogens including *Brucella abortus* [32], *Yersinia enterocolitica* [33], *Salmonella enterica* [34], *Legionella pneumophila* [13], *Shigella flexneri* [35], *Klebsiella pneumoniae* [14], *Listeria monocytogenes* [36], *Burkholderia cenocepacia* [17], *Chlamydia trachomatis* [37], *Borrelia burgdorferi* [23], *Mycobacterium tuberculosis* [38] and *Haemophilus parasuis* [39]. In contrast, the deletion of the *htrA* gene in *H. pylori* has not yet been reported, and the generation of Δ*htrA* knockout mutants was found to be lethal [40, 41]. Given the fact that *H. pylori htrA* is an essential bifunctional gene with crucial intracellular and extracellular functions, it may be justified to consider HtrA as a new target for future anti-bacterial therapy.

### Why is HtrA inhibition a step forward in the fight against pathogens?

With the exception of *Mycoplasma genitalium* and *Methanococcus janaschii*, it seems that all bacterial pathogens and commensals in the microbiota express HtrA proteins; a fact that evades the classical and precise definition of virulence or pathogenic factors [42]. Consequently, this observation leads to the question if such a factor might also serve as a potent macromolecular drug target? In fact, targeting HtrA offers some potential advantages:

(i.) it is secreted into the extracellular micro-milieu or presented on the bacterial cell surface and therefore accessible to drug compounds [43, 44],
(ii.) it has a defined enzymatic active site and substrate recognition [19, 20, 45, 46],
(iii.) it cleaves E-cadherin, proteoglycans and fibronectin as host factors with important functions for bacterial pathogenesis [19–21, 47], and
(iv.) it is an essential enzyme in *H. pylori* physiology [40, 41].

These characteristics make HtrA a potentially attractive candidate for novel therapeutic approaches to treat bacterial pathogenesis.

**Fig. 2:**
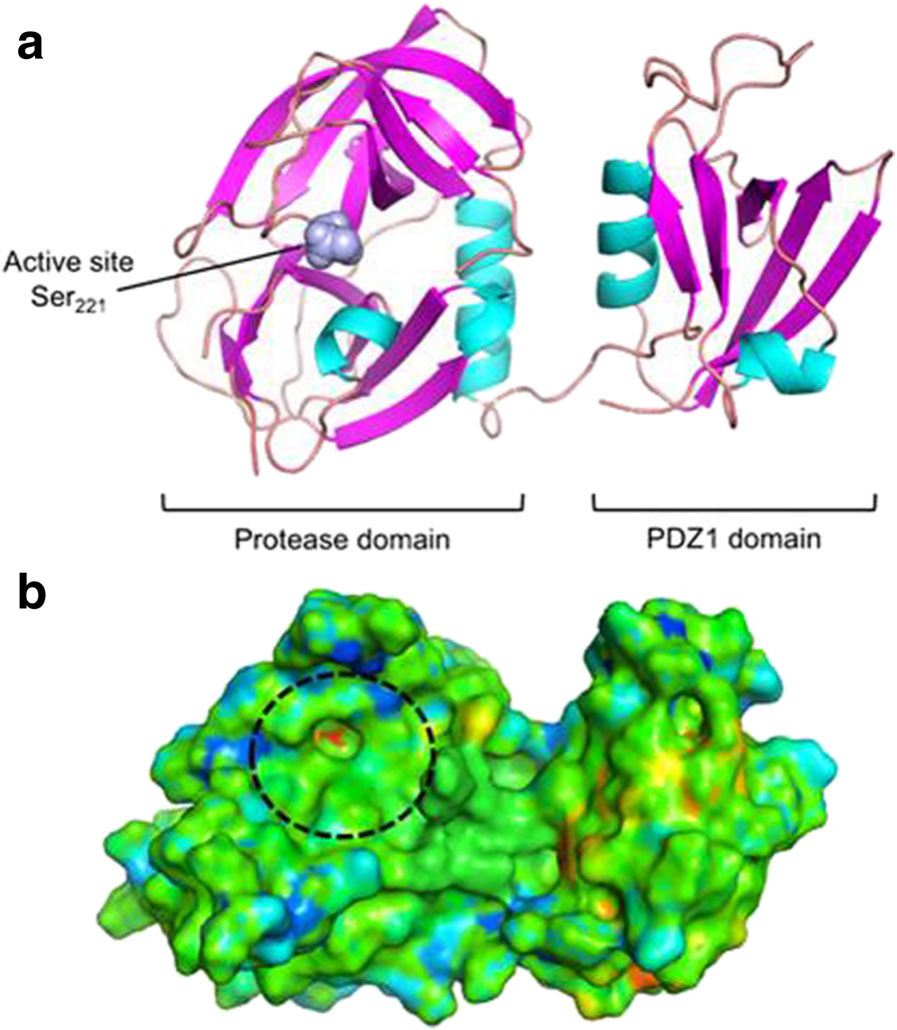
Structural model of the *H.pylori* HtrA monomer. The model is based on a preliminary X-ray crystal structure of the *apo*-enzyme containing one of the two PDZ domains [63]. The cartoon structure (**a**) shows the protease domain with the catalytic residue Ser_221_ highlighted. The interface between the protease domain and PDZ1 is mediated by helix-helix interactions. The surface representation (**b**) has the same orientation as in (**a**). Temperature coloring is according to the computed “ligandability” [45, 64]. A potential ligand interaction “hot spot” is predicted inside the active site (approximated by the *dashed circle*). This model and related computational analyses support the design of *H. pylori* HtrA inhibitors. The graphics were prepared with MacPyMol (v1.7, Schrödinger LLC, New York, NY, USA)

The current model of HtrA function in bacterial pathogenesis is based on the hypothesis that HtrA-mediated E-cadherin cleavage represents a central step in bacterial pathogenesis prior to and/or after the interference of virulence factors (e.g., effector proteins, cytotoxins, adhesins) with the integrity of the polarized epithelium [48, 49]. These complex pathogen-host interactions require sophisticated and coordinated mechanisms to provide access to laterally expressed E-cadherin and subsequently to basolaterally presented host cell receptors or circulating cells of the immune system in deeper regions of the tissues. In principle, the opening of tight junctions has been shown to be HtrA-independent in *H. pylori* [20] and *C. jejuni* [21], indicating that additional bacterial factors are involved in the disruption of the epithelial polarity. In *H. pylori* infections, soluble factors such as vacuolating cytotoxin A (VacA), cytotoxin-associated gene A (CagA) and urease were previously described to open up tight junctions [50–52], underlining that the interplay of various pathogenic factors and HtrA is responsible for disrupting the lateral junctions between epithelial cells. The mechanism by which *C. jejuni* opens tight junctions is yet unknown. For both pathogens, an HtrA-mediated transmigration process was observed [20, 21, 28], enabling bacterial contact with basolaterally expressed receptors, such as α5β1 integrins or fibronectin [53, 54], but also allowing the bacteria to directly interact with cells of the immune system. It is currently being investigated whether *C. jejuni* prefers the transcellular migration or paracellular route, or whether this pathogen combines two pathways to overcome the epithelial barrier [48]. However, HtrA-mediated E-cadherin cleavage in concert with activated host proteases has been shown to promote pathogenesis in vitro for *H. pylori* [20, 55, 56] and in *C. jejuni* animal models [30, 31], which has been summarized in several review articles [49, 57]. Beta1-integrins and fibronectin have already been identified as important binding partners for a number of additional pathogens including *Yersinia pseudotuberculosis, Staphylococcus aureus*, *Klebsiella pneumoniae*, and others [58], indicating the importance of opening intercellular adhesion complexes. The observation that additional gastrointestinal pathogens (*Shigella flexneri*, enteropathogenic *Escherichia coli* [EPEC], *Yersinia enterocolitica*, *Salmonella enterica* sub. Enterica) utilize the HtrA homologs DegP and DegQ for E-cadherin cleavage during infection of cultured epithelial cells and in vitro underlines a function of HtrA proteins as “virulence- or pathogenicity-promoting” factors [19, 59]. Based on this hypothesis, it is enticing to surmise that pharmacological inhibitors blocking extracellular HtrA activity could stop bacterial transmigration and tissue invasion in vivo, while leaving the microbiota unaffected. Consequently, selective pharmacological inhibition of HtrA might facilitate antibiotic treatment by preventing bacterial access to deeper regions of gastrointestinal tissues. Possibly, bacterial HtrAs could also target additional substrates. For *Chlamydia trachomatis*, it was demonstrated that HtrA is secreted into the chlamydia-containing vesicles and into the host cytoplasm. Although substrates for HtrA were not identified, inhibition of HtrA efficiently affected the bacterial life cycle and survival [60, 61]. With the availability of high-resolution structural models of the various HtrAs from relevant pathogens, structure-based inhibitor design should become feasible (Fig. 2).

In contrast to other investigated bacterial species, *H. pylori* HtrA synthesis appears to be crucially important for bacterial physiology and survival since any intervention via mutagenesis or deletion of the *htrA* gene in the genome of *H. pylori* has not been successful up to date [20, 40, 41]. Correspondingly, a naturally occurring *htrA*-negative *H. pylori* isolate was not found in a comprehensive screening of more than 990 samples [41]. These observations point to the question whether pharmacological inhibition of HtrA could tackle *H. pylori* physiology specifically? *Helicobacter* HtrA inhibitor (HHI) was the first described small molecule compound inhibiting *H. pylori* HtrA [20], which blocked HtrA-mediated E-cadherin cleavage and subsequent bacterial transmigration across a polarized epithelial monolayer. However, HHI did not affect the bacterial survival [20] and it is unknown, whether HHI is actually taken up by the bacteria. A first step in the direction of a future targeted *H. pylori* therapy has recently been made by demonstrating that compound 1 drastically affected *H. pylori* survival and/or growth [41, 62]. The data obtained suggest that compound 1 penetrates the bacterial cell wall to block periplasmic HtrA activity and subsequently *H. pylori* survival. Further research will be necessary to identify and optimize small molecule HtrA inhibitors as anti-*H. pylori* pharmacological lead compounds.

## Conclusions

New strategies are urgently needed to combat bacterial infections. At the first glance, targeting a widespread bacterial enzyme does not appear to be straightforward. However, considering the HtrA-mediated host cell factor processing as a central step in the pathogenesis of many different infectious bacteria opens up a new perspective. Inhibiting extracellular HtrA by compounds that do not penetrate the bacterial membrane will likely not affect the colonization and survival of commensals; thus solely interference of pathogens with their individual virulence/pathogenic factors with the epithelium will be limited. Potent HtrA inhibitors penetrating the periplasm of *H. pylori* might pave the way towards a targeted anti-*H. pylori* treatment owed to the fact that *H. pylori* physiology essentially requires functional HtrA activity. While many of the current antibiotics affect all bacteria independently of assets and drawbacks for the colonized host, pathogen-selective HtrA inhibitors might present a drug discovery opportunity.

## Abbreviations

DegP/Q: Periplasmic serine endoproteases
HtrA: High temperature requirement A
PDZ: Postsynaptic density protein (PSD95), *Drosophila* disc large tumor suppressor (Dlg1), and zonula occludens-1 protein (ZO-1).

## References

[1] Kim DY, Kim KK (2005). Structure and function of HtrA family proteins, the key players in protein quality control. J Biochem Mol Biol.

[2] Ingmer H, Brondsted L (2009). Proteases in bacterial pathogenesis. Res Microbiol.

[3] Clausen T, Kaiser M, Huber R, Ehrmann M (2011). HTRA proteases: regulated proteolysis in protein quality control. Nat Rev Mol Cell Biol.

[4] Frees D, Brondsted L, Ingmer H (2013). Bacterial proteases and virulence. Subcell Biochem.

[5] Gottesman S, Maurizi MR, Wickner S (1997). Regulatory subunits of energy-dependent proteases. Cell.

[6] Singh N, Kuppili RR, Bose K (2011). The structural basis of mode of activation and functional diversity: a case study with HtrA family of serine proteases. Arch Biochem Biophys.

[7] Skorko-Glonek J, Zurawa-Janicka D, Koper T, Jarzab M, Figaj D, Glaza P, Lipinska B (2013). HtrA protease family as therapeutic targets. Curr Pharm Des.

[8] Hansen G, Hilgenfeld R (2013). Architecture and regulation of HtrA-family proteins involved in protein quality control and stress response. Cell Mol Life Sci.

[9] Jiang J, Zhang X, Chen Y, Wu Y, Zhou ZH, Chang Z, Sui SF (2008). Activation of DegP chaperone-protease via formation of large cage-like oligomers upon binding to substrate proteins. Proc Natl Acad Sci U S A.

[10] Krojer T, Sawa J, Schafer E, Saibil HR, Ehrmann M, Clausen T (2008). Structural basis for the regulated protease and chaperone function of DegP. Nature.

[11] Bass S, Gu Q, Christen A (1996). Multicopy suppressors of prc mutant Escherichia coli include two HtrA (DegP) protease homologs (HhoAB), DksA, and a truncated R1pA. J Bacteriol.

[12] Lipinska B, Fayet O, Baird L, Georgopoulos C (1989). Identification, characterization, and mapping of the Escherichia coli htrA gene, whose product is essential for bacterial growth only at elevated temperatures. J Bacteriol.

[13] Pedersen LL, Radulic M, Doric M, Abu Kwaik Y (2001). HtrA homologue of Legionella pneumophila: an indispensable element for intracellular infection of mammalian but not protozoan cells. Infect Immun.

[14] Cortes G, de Astorza B, Benedi VJ, Alberti S (2002). Role of the htrA gene in Klebsiella pneumoniae virulence. Infect Immun.

[15] Brondsted L, Andersen MT, Parker M, Jorgensen K, Ingmer H (2005). The HtrA protease of Campylobacter jejuni is required for heat and oxygen tolerance and for optimal interaction with human epithelial cells. Appl Environ Microbiol.

[16] Mo E, Peters SE, Willers C, Maskell DJ, Charles IG (2006). Single, double and triple mutants of Salmonella enterica serovar Typhimurium degP (htrA), degQ (hhoA) and degS (hhoB) have diverse phenotypes on exposure to elevated temperature and their growth in vivo is attenuated to different extents. Microb Pathog.

[17] Flannagan RS, Aubert D, Kooi C, Sokol PA, Valvano MA (2007). Burkholderia cenocepacia requires a periplasmic HtrA protease for growth under thermal and osmotic stress and for survival in vivo. Infect Immun.

[18] Boehm M, Lind J, Backert S, Tegtmeyer N (2015). Campylobacter jejuni serine protease HtrA plays an important role in heat tolerance, oxygen resistance, host cell adhesion, invasion, and transmigration. Eur J Microbiol Immunol.

[19] Klenner A, Hahnke V, Geppert T, Schneider P, Zettl H, Haller S, Rodrigues T, Reisen F, Hoy B, Schaible AM (2012). From virtual screening to bioactive compounds by visualizing and clustering of chemical space. Molecular informatics.

[20] Hoy B, Lower M, Weydig C, Carra G, Tegtmeyer N, Geppert T, Schroder P, Sewald N, Backert S, Schneider G (2010). Helicobacter pylori HtrA is a new secreted virulence factor that cleaves E-cadherin to disrupt intercellular adhesion. EMBO Rep.

[21] Boehm M, Hoy B, Rohde M, Tegtmeyer N, Baek KT, Oyarzabal OA, Brondsted L, Wessler S, Backert S (2012). Rapid paracellular transmigration of Campylobacter jejuni across polarized epithelial cells without affecting TER: role of proteolytic-active HtrA cleaving E-cadherin but not fibronectin. Gut pathogens.

[22] Elmi A, Nasher F, Jagatia H, Gundogdu O, Bajaj-Elliott M, Wren B, Dorrell N (2016). Campylobacter jejuni outer membrane vesicle-associated proteolytic activity promotes bacterial invasion by mediating cleavage of intestinal epithelial cell E-cadherin and occludin. Cell Microbiol.

[23] Coleman JL, Crowley JT, Toledo AM, Benach JL (2013). The HtrA protease of Borrelia burgdorferi degrades outer membrane protein BmpD and chemotaxis phosphatase CheX. Mol Microbiol.

[24] Altindis E, Fu Y, Mekalanos JJ (2014). Proteomic analysis of Vibrio cholerae outer membrane vesicles. Proc Natl Acad Sci U S A.

[25] Bartolini E, Ianni E, Frigimelica E, Petracca R, Galli G, Berlanda Scorza F, Norais N, Laera D, Giusti F, Pierleoni A, et al. Recombinant outer membrane vesicles carrying Chlamydia muridarum HtrA induce antibodies that neutralize chlamydial infection in vitro. J Extracell Vesicles. 2013;2. 10.3402/jev.v2i0.20181 PMC3760637 24009891

[26] Olofsson A, Vallstrom A, Petzold K, Tegtmeyer N, Schleucher J, Carlsson S, Haas R, Backert S, Wai SN, Grobner G (2010). Biochemical and functional characterization of Helicobacter pylori vesicles. Mol Microbiol.

[27] Niessen CM (2007). Tight junctions/adherens junctions: basic structure and function. J Invest Dermatol.

[28] Schmidt TP, Perna AM, Fugmann T, Bohm M, Jan H, Haller S, Gotz C, Tegtmeyer N, Hoy B, Rau TT (2016). Identification of E-cadherin signature motifs functioning as cleavage sites for Helicobacter pylori HtrA. Sci Rep.

[29] Schmidt TP, Goetz C, Huemer M, Schneider G, Wessler S (2016). Calcium binding protects E-cadherin from cleavage by Helicobacter pylori HtrA. Gut pathogens.

[30] Heimesaat MM, Alutis M, Grundmann U, Fischer A, Tegtmeyer N, Bohm M, Kuhl AA, Gobel UB, Backert S, Bereswill S (2014). The role of serine protease HtrA in acute ulcerative enterocolitis and extra-intestinal immune responses during Campylobacter jejuni infection of gnotobiotic IL-10 deficient mice. Front Cell Infect Microbiol.

[31] Heimesaat MM, Fischer A, Alutis M, Grundmann U, Boehm M, Tegtmeyer N, Gobel UB, Kuhl AA, Bereswill S, Backert S (2014). The impact of serine protease HtrA in apoptosis, intestinal immune responses and extra-intestinal histopathology during Campylobacter jejuni infection of infant mice. Gut pathogens.

[32] Elzer PH, Phillips RW, Kovach ME, Peterson KM, Roop RM (1994). Characterization and genetic complementation of a Brucella abortus high-temperature-requirement A (htrA) deletion mutant. Infect Immun.

[33] Li SR, Dorrell N, Everest PH, Dougan G, Wren BW (1996). Construction and characterization of a Yersinia enterocolitica O:8 high-temperature requirement (htrA) isogenic mutant. Infect Immun.

[34] Humphreys S, Stevenson A, Bacon A, Weinhardt AB, Roberts M (1999). The alternative sigma factor, sigmaE, is critically important for the virulence of Salmonella typhimurium. Infect Immun.

[35] Purdy GE, Hong M, Payne SM (2002). Shigella flexneri DegP facilitates IcsA surface expression and is required for efficient intercellular spread. Infect Immun.

[36] Wilson RL, Brown LL, Kirkwood-Watts D, Warren TK, Lund SA, King DS, Jones KF, Hruby DE (2006). Listeria monocytogenes 10403S HtrA is necessary for resistance to cellular stress and virulence. Infect Immun.

[37] Gloeckl S, Ong VA, Patel P, Tyndall JD, Timms P, Beagley KW, Allan JA, Armitage CW, Turnbull L, Whitchurch CB (2013). Identification of a serine protease inhibitor which causes inclusion vacuole reduction and is lethal to Chlamydia trachomatis. Mol Microbiol.

[38] Roberts DM, Personne Y, Ollinger J, Parish T (2013). Proteases in Mycobacterium tuberculosis pathogenesis: potential as drug targets. Future Microbiol.

[39] Zhang L, Li Y, Wen Y, Lau GW, Huang X, Wu R, Yan Q, Huang Y, Zhao Q, Ma X (2016). HtrA is important for stress resistance and virulence in haemophilus parasuis. Infect Immun.

[40] Salama NR, Shepherd B, Falkow S (2004). Global transposon mutagenesis and essential gene analysis of Helicobacter pylori. J Bacteriol.

[41] Tegtmeyer N, Moodley Y, Yamaoka Y, Pernitzsch SR, Schmidt V, Traverso FR, Schmidt TP, Rad R, Yeoh KG, Bow H (2016). Characterisation of worldwide Helicobacter pylori strains reveals genetic conservation and essentiality of serine protease HtrA. Mol Microbiol.

[42] Lu H, Yamaoka Y, Graham DY (2005). Helicobacter pylori virulence factors: facts and fantasies. Curr Opin Gastroenterol.

[43] Boehm M, Haenel I, Hoy B, Brondsted L, Smith TG, Hoover T, Wessler S, Tegtmeyer N (2013). Extracellular secretion of protease HtrA from Campylobacter jejuni is highly efficient and independent of its protease activity and flagellum. Eur J Microbiol Immunol.

[44] Lower M, Weydig C, Metzler D, Reuter A, Starzinski-Powitz A, Wessler S, Schneider G (2008). Prediction of extracellular proteases of the human pathogen Helicobacter pylori reveals proteolytic activity of the Hp1018/19 protein HtrA. PLoS One.

[45] Geppert T, Hoy B, Wessler S, Schneider G (2011). Context-based identification of protein-protein interfaces and “hot-spot” residues. Chem Biol.

[46] Lower M, Geppert T, Schneider P, Hoy B, Wessler S, Schneider G (2011). Inhibitors of Helicobacter pylori protease HtrA found by ‘virtual ligand’ screening combat bacterial invasion of epithelia. PLoS One.

[47] Gherardini FC (2013). Borrelia burgdorferi HtrA may promote dissemination and irritation. Mol Microbiol.

[48] Backert S, Boehm M, Wessler S, Tegtmeyer N (2013). Transmigration route of Campylobacter jejuni across polarized intestinal epithelial cells: paracellular, transcellular or both?. Cell Commun Signal.

[49] Posselt G, Backert S, Wessler S (2013). The functional interplay of Helicobacter pylori factors with gastric epithelial cells induces a multi-step process in pathogenesis. Cell Commun Signal.

[50] Wroblewski LE, Shen L, Ogden S, Romero-Gallo J, Lapierre LA, Israel DA, Turner JR, Peek RM (2009). Helicobacter pylori dysregulation of gastric epithelial tight junctions by urease-mediated myosin II activation. Gastroenterology.

[51] Lytton SD, Fischer W, Nagel W, Haas R, Beck FX (2005). Production of ammonium by Helicobacter pylori mediates occludin processing and disruption of tight junctions in Caco-2 cells. Microbiology.

[52] Papini E, Satin B, Norais N, de Bernard M, Telford JL, Rappuoli R, Montecucco C (1998). Selective increase of the permeability of polarized epithelial cell monolayers by Helicobacter pylori vacuolating toxin. J Clin Invest.

[53] Kwok T, Zabler D, Urman S, Rohde M, Hartig R, Wessler S, Misselwitz R, Berger J, Sewald N, Konig W (2007). Helicobacter exploits integrin for type IV secretion and kinase activation. Nature.

[54] Jimenez-Soto LF, Kutter S, Sewald X, Ertl C, Weiss E, Kapp U, Rohde M, Pirch T, Jung K, Retta SF (2009). Helicobacter pylori type IV secretion apparatus exploits beta1 integrin in a novel RGD-independent manner. PLoS Pathog.

[55] Schirrmeister W, Gnad T, Wex T, Higashiyama S, Wolke C, Naumann M, Lendeckel U (2009). Ectodomain shedding of E-cadherin and c-Met is induced by Helicobacter pylori infection. Exp Cell Res.

[56] Costa AM, Ferreira RM, Pinto-Ribeiro I, Sougleri IS, Oliveira MJ, Carreto L, Santos MA, Sgouras DN, Carneiro F, Leite M (2016). Helicobacter pylori Activates Matrix Metalloproteinase 10 in Gastric Epithelial Cells via EGFR and ERK-mediated Pathways. J Infect Dis.

[57] Wessler S, Backert S (2008). Molecular mechanisms of epithelial-barrier disruption by Helicobacter pylori. Trends Microbiol.

[58] Ulanova M, Gravelle S, Barnes R (2009). The role of epithelial integrin receptors in recognition of pulmonary pathogens. J Innate Immun.

[59] Abfalter CM, Schubert M, Götz C, Schmidt TP, Posselt G, Wessler S (2016). HtrA-mediated E-cadherin cleavage is limited to DegP and DegQ homologs expressed by gram-negative pathogens. Cell Commun Signal.

[60] Patel P, De Boer L, Timms P, Huston WM (2014). Evidence of a conserved role for Chlamydia HtrA in the replication phase of the chlamydial developmental cycle. Microbes Infect.

[61] Wu X, Lei L, Gong S, Chen D, Flores R, Zhong G (2011). The chlamydial periplasmic stress response serine protease cHtrA is secreted into host cell cytosol. BMC Microbiol.

[62] Perna AM, Rodrigues T, Schmidt TP, Bohm M, Stutz K, Reker D, Pfeiffer B, Altmann KH, Backert S, Wessler S (2015). Fragment-based De novo design reveals a small-molecule inhibitor of helicobacter pylori HtrA. Angewandte Chemie (International ed in English).

[63] Perna AM, Reisen F, Schmidt TP, Geppert T, Pillong M, Weisel M, Hoy B, Simister PC, Feller SM, Wessler S (2014). Inhibiting Helicobacter pylori HtrA protease by addressing a computationally predicted allosteric ligand binding site. Chem Sci.

[64] Todoroff N, Kunze J, Schreuder H, Hessler G, Baringhaus KH, Schneider G (2014). Fractal dimensions of macromolecular structures. Mol Inform.

